# Supplementary material for: Syllable processing is organized in discrete subregions of the human superior temporal gyrus
Source: PLoS Biol. 2024 Sep 6;22(9):e3002774. doi: 10.1371/journal.pbio.3002774 (PMC11410217; doi:10.1371/journal.pbio.3002774)
Supplement: Attachment — Submitted filename: Dan PLOS Modular Response to Reviews 4.docx [file pbio.3002774.s015.docx]

REVIEWS:

Reviewer #1: SUMMARY

The authors used microarrays with very fine-grained spatial resolution. They recorded neural responses in superior temporal gyrus from 7 patients while they listened to consonant-vowel syllables in clear and noisy (via noise vocoding) conditions. They analyzed the similarity of responses from electrodes at variable distances in order to test for and describe the modular structure that emerges. They found that responses within ~1.7mm "modules" were very similar, which transition to a different response in the neighboring modules. This was replicated when using a number of different analysis methods, including supervised and non-supervised approaches. The authors conclude that the similarity structure they observe provides evidence for discrete processing modules along STG, with a consistent size, shape and density across patient recordings.

Overall, this is important research that I think will help propel human neuroscience into a new era of spatially resolved recordings and important discoveries.

I noted some weaknesses that need to be addressed before I can fully endorse this article for publication.

MAJOR

[1] A big claim of the current paper is in identifying the size and shape of "processing modules". I would like to see some sanity checks that the design of the micro-array itself would not be biased to finding auto-correlation structure of that reported in the study. This could be achieved by analyzing the auto-correlation structure in the noise of the micro-arrays, and conducting dummy-recordings on agar or similar.

*It is extremely unlikely that Phase-Locking Values (PLV) greater than 0.8 (which were typically observed within modules) would arise from the design of the microgrid itself. The contacts are in a regular grid to a very high level of precision and so variable proximity cannot account for the module assignments. We explicitly tested if the modules identified by NNMF could be due to cross-talk in the transit from the electrode to the electronics, at the level of the ADCs, or in shared processing characteristics in the electronics (Supplementary Fig. 8). First, we plotted a histogram of the physical distances between contacts belonging to the same module (Suppl. Fig. 8A). As expected, given that modules are compact spatially, these formed a roughly Gaussian distribution centered at ~1mm and never exceeding ~2.3mm. In contrast, the histogram of proximities of the wires of connecting contacts belonging to the same module had two main peaks, one at very short distances mainly ~1-8 separations, and one at ~800-1000 separations (Suppl. Fig. 8BC). Thus, these long distances between wires carrying signals with phase-specific similarities cannot be accounted for by simple crosstalk between wires.*

*The wires carry signals to a total of 16 individual analog-to-digital converters (ADC), with each ADC receiving 64 input from 64 contact. The organizational pattern of electrodes to ADCs is shown in Suppl. Fig 8D; a similar mosaic pattern of alternating rows is not appreciated in any of the modules identified. If the shared ADC processing caused the module clustering, individual clusters should then also have a mosaic of members on alternate rows, which they did not.*

*Note also that the NNMF procedure is completely agnostic to the spatial organization of the contacts; thus if cross-talk in the ribbon cable, or shared amplifier bank characteristics underlay the modules, then they would have reproduced the proximity matrices of the wires or of the ADC assignments, rather than being organized in spatially-compact roughly circular domains that span multiple wire and ADC domains. We also note that the same ribbon-cable and ADC bank assignments were used in all experiments and yet quite different module locations were observed. Furthermore, in one patient, the same actual array was simply moved from one STG site to another between blocks of the task, resulting in different module locations being observed on the same array. This evidence is inconsistent with modules arising from technical sources of signal similarity. Finally, the similarity of responses within modules with sharp demarcations in latency between modules are visible in traversals of the grid (Figs. 3BF) which could not be due to technical properties of the grids.*

[2] Another claim here is that the authors find "phoneme-selectivity". However, the authors conduct no analyses that support this claim directly. Phase-locking is indirectly related to the fact that the consonants occurred before the vowels across all stimuli. But a delayed response to a consonant and an immediate response to a vowel could result in the same phase-locking value. If the authors would like to make a claim about phonetic encoding, I suggest that they provide an analysis of the responses based on the factorial design of their stimuli {plosive, fricative, liquid, nasal} consonants and {low, medium, high} vowels.

*We apologize for the miscommunication. Indeed we did use the term “phoneme-selectivity” in the Abstract but in the text we make clear that we are referring to selectivity for phonemes as compared to noise-vocoded controls. We have replaced “phoneme-selectivity” with “stimulus-selectivity” in the Abstract.*

*We did categorize our stimuli as the reviewer suggests, but unfortunately, the acute intraoperative environment did not permit a sufficient number of trials to characterize our responses in that manner, but only to show that the responses were selective for phonemes as compared to noise-vocoded matched controls. We have further emphasized that distinction in our revised manuscript.*

[3] The patients hear CV syllables, which will sometimes form valid English words (e.g., "seɪ") and sometimes will not (e.g., "zeɪ"). The task of the participants is to press a button when they recognize a word of English, but the authors analyze responses to all CV syllables, not just those that are valid words. I ask the authors to clarify in the manuscript that analyses were performed on clear syllables and noise-vocoded syllables, where some items were words and some were not. Furthermore, to change the corresponding labels on the figures, and change them from "words" to "syllables" — or, similar.

*We have made the suggested changes. “Word” was replaced with “Syllable” wherever possible, and we added the phrase “(words and pseudo-words were combined for analysis).”*

[4] There were a few sentences in the abstract that I found unclear. I request that the following are re-phrased to be clearer what is meant:

"modular organization fundamental to cortical processing" — at what spatial scale are you referring to here?

*Changed to “Modular organization at ~1mm scale could be fundamental…”*

"its presence in human association cortex is unknown" — if modular organization is critical, then how can its presence be unknown? Again, what spatial scale is being referred to?

*See just above.*

"characterized phoneme processing" — relating to point (2) above, I don't agree that this is an accurate summary of what was characterized in this study

*Changed to "characterized syllable processing".*

"phoneme selectivity" — see point (2) above

*Changed to "stimulus-selectivity".*

MINOR

In Figure 1C, it is not clear to me what is the array, and what is the shadow of another object.

*We made the outline of the array much more salient.*

In Figure 1D, why are single electrode positions visualized, when the authors use an entire array?

*The circles in 1D are ~5mm in diameter which is comparable in size to the arrays (0.2x3.2mm for the 128 ch array and 3.2x12.8mm for the 1024 ch grid. Since this is a standard brain, locations are approximate. We rephrase the legend as follows: “****D.*** *Approximate centers of electrode arrays on standard brain.”*

In Figure 3E and elsewhere, why are there "empty" regions where no modules emerge?

*There are many reasons this may arise. The small gaps are typically due to contacts that are not functional for technical reasons. The large gaps are typically due to surgically-motivated limitations in placement resulting in part of the array being not in contact with the pial surface (e.g., the left side in Fig. 3E).*

Reviewer #2 (Yves Boubenec): In this work, the authors tested the putative existence of functional modules within human auditory cortex. They show that functional responses are shared within regions of a couple of mm in diameter, suggesting discrete processing modules. That is a fascinating results that echo with long-standing literature of columnar processing in visual cortex.

The study is concise and compact, which is pleasant, but makes it missing a few points for clarity. Little, if none, is said about phonetic/syllabic tuning for these cortical domains.

________________

major comments:

From figure 3, it looks like the factorization algorithm grouped channels mainly by the temporal profile of the response time-courses. This is indeed an important feature of functional responses. Nevertheless, these columns show strong speech responses, and one should not ignore the tuning of these channels.

1) Does the clustering depend on using the time-course? Or are similar results found when using the average response to each sound (ignoring the time-course)?

*We have added the following sentence to the description of the NNMF: “The entire unaveraged signal from all technically-correct channels over the entire task was entered into the factorization.” We suspect that using the averaged response across trials will be as effective in identifying the modules because it is their distinct responses across trials that characterizes them.*

2) As a matter of fact, I could not find much detail in the methods about the actual data provided to the factorization algorithm. Does it simply perform on the average response time-course? On the average time-course for noise and syllable stimuli? On the average time-course for each syllable? Or on the single-trial responses?

*Please see response to previous query.*

3) A clear link with previous literature is missing. What is the phonetic tuning of these modules? Is it consistent with results of Mesgarani's and Chang's teams (cited in the introduction)? Do they reveal another level of organization that previous studies could have missed (like each module being heavily dedicated to one specific group of phonemes)?

An alternative option is that, these modules could be poorly tuned to phonemes themselves, but instead exhibit syllabic tuning. And the data of this study can address this issue.

*As we point out in the text, previous studies of the STG response to speech (Mesgarani et al and Leonard et al, in particular) have not found HG responses that were selective for particular phonemes, but rather to spectro-temporal patterns that were characteristic of different categories of speech sounds. We examined the responses to our stimuli in an attempt to identify similar specificity in our modules, but unfortunately, the acute intraoperative environment did not permit a sufficient number of trials to characterize our responses in that manner, but only to show that the responses were selective for CV syllables as compared to noise-vocoded matched controls. However, for the purposes of the current study this was adequate because our goal was to determine the spatial distribution of physiological responses to a variety of phonemic stimuli, which we accomplished. We expect to receive FDA clearance to use similar microgrids in the semichronic setting and that will allow us to systematically explore their phonetic tuning.*

________________

minor comments:

i) I was unclear about the exact stimulus set: do you confirm there are 24 distinct syllables in total?

*We clarified the task description as follows: “Eight consonants were drawn equally from 4 categories following linguistic categories: Obstruent-Plosive (d, g), Obstruent-Fricative (s, z), Sonorant-Liquid (l, r), and Sonorant-Nasal (m, n). Nine vowels are drawn equally from 3 categories: Type 1 (u, aɪ, ɔ), Type 2 (ə, I, ɛ), and Type 3 (eɪ, æ, aʊ). Each possible CV combination is equally represented in the total stimulus set, resulting in 72 total syllables.”*

ii) figures 2 IJK are not referenced. Reference to figure 2H refers in fact to figure 2K.

*Corrected*

iii) my understanding is that no actual word was played. Instead, stimuli were single syllables. I suggest replacing 'words' in the figures by 'syllables'.

*Corrected*

iv) acronym PLV (for phase locking) in the caption comes out of the blue.

*Corrected*
