# Supplementary material for: Syllable processing is organized in discrete subregions of the human superior temporal gyrus
Source: PLoS Biol. 2024 Sep 6;22(9):e3002774. doi: 10.1371/journal.pbio.3002774 (PMC11410217; doi:10.1371/journal.pbio.3002774)
Supplement: Attachment — Submitted filename: Cleary Modular Response to Second Review v5.docx [file pbio.3002774.s016.docx]

Dear Professor Schnell,

REVIEWS:

*Reviewer #1: The authors have addressed my concerns - thank you.*

Thank you!

*Reviewer #2 (Yves Boubenec): I thank the authors you for their response and the revisions made to the manuscript. However, I remain concerned that my main critique has not been fully addressed. Specifically, there is still no analysis demonstrating that the NNMF leverages differences in responses across phonemes rather than primarily grouping channels based on generic temporal response patterns (e.g., onset or sustained responses).*

*To adequately address this point, I propose redoing the analysis using the time-averaged response (ignoring the time-course) for each sound. This would help determine whether the clustering observed is truly due to differences in phoneme-specific responses or if it is primarily driven by the temporal characteristics of the responses. Demonstrating that similar modules are identified using time-averaged responses would strengthen the argument that the NNMF is capturing phoneme-specific tuning rather than generic temporal response patterns.*

*Without this analysis, it remains unclear whether the observed clustering reflects phoneme-specific processing or if it is an artifact of the temporal dynamics of the neural responses. Therefore, I strongly recommend conducting this additional analysis to address this critical aspect of the study.*

Thank you for your comment, we appreciate your help in making this a stronger manuscript.

We have performed the requested analysis, and show that similar modules are identified when only a very limited data set is used (the time-averaged responses from 100 – 400 ms in each trial, instead of the entire recording). Two examples are shown in Supp. Figure 9, comparing the time-averaged vs original analysis for Pt. 6A (panel C vs. D) and Pt. 6B (panel G vs. H). Please note that the time-averaged analysis entered only ~200 samples for each of the ~1000 channels (one for each trial), whereas the original method entered ~300k samples per channel (~5min at 1000Hz). Inasmuch as factorial analyses of this kind are well-known to require a large number of samples it is likely that the differences between the panels are due to both the decreased data entering the time-averaged NNMF as well as the specific absence of temporal information.

In Pt. 6A, the time courses of original modules 2 and 4 are overlapping but peak at different times (Suppl. Fig. 6F), and they are poorly differentiated in the time-averaged NNMF (Suppl. Fig. 9C), suggesting that the time courses of the response did contribute to the delineation of these modules. However, note that even in these cases, the time-courses of the modules were not indicative of classical auditory “on” versus “off”, or “sustained” versus “phasic” responses.

In Pt. 6B, all modules have very similar time courses (Suppl. Fig. 7CD) and thus module selection by the original method is unlikely to reflect differences in temporal response patterns. Similar temporal response patterns between distinct modules are also seen in other patients, for example Patient 4 (a linear array shown in Supplementary figure 1).

We conclude that while it is possible that time-course differences made some contribution to refining the boundaries of the modules we present, such differences are not generic auditory on-off/sustained-phasic response modulations but rather modulation within the CV syllable as would be expected of a module differentially responding to the spectro-temporal patterns characterizing different phoneme classes. Thus, such differences would constitute part of the functional phoneme-selective response profile of the module. We now note this in the text.

Finally, we would also like to note that we are careful to refer to the responses as ‘phoneme selective’ rather than ‘phoneme specific’. This is clearly established by the significantly different responses to syllables vs noise in 19 of the 27 modules which were performed on the average high gamma from 50-450ms on each trial, and thus are not a simple consequence of the “generic temporal response patterns,” i.e., the time-course of the response to all stimuli at the studied locations.
